# Identification of a novel compound heterozygous *IDUA* mutation underlies Mucopolysaccharidoses type I in a Chinese pedigree

**DOI:** 10.1002/mgg3.1058

**Published:** 2019-11-23

**Authors:** Yong‐An Zhou, Ping Li, Yanping Zhang, Qiuhong Xiong, Chao Li, Zhonghua Zhao, Yuxian Wang, Han Xiao

**Affiliations:** ^1^ Bluttransfusion The Second Hospital Shanxi Medical University Shanxi Taiyuan China; ^2^ Institutes of Biomedical Sciences Shanxi University Taiyuan China; ^3^ Department of Obstetrics and Gynecology The First Hospital Shanxi Medical University Taiyuan China

**Keywords:** IDUA, lysosomal storage disease, MPS I, WES

## Abstract

**Background:**

Mucopolysaccharidosis type I (MPS I) is a rare autosomal storage disorder resulting from the defective alpha‐L‐iduronidase (encoded by *IDUA*) enzyme activity and accumulation of glycosaminoglycans (GAGs) in lysosomes. So far, more than 100 *IDUA* causative mutations have been identified leading to three MPS I phenotypic subtypes: Hurler syndrome (severe form), Hurler/Scheie syndrome (intermediate form), and Scheie syndrome (mild form).

**Methods:**

Whole‐exome sequencing (WES) was performed to identify the underlying genetic mutations. To verify the identified variations, Sanger sequencing was performed for all available family members following PCR amplification. The impact on IDUA protein was analyzed by sequential analysis and homology modeling.

**Results:**

A novel *IDUA* heterozygous single base insertion (c.1815dupT, p.V606Cfs51^*^) and a known missence mutation (c.T1037G, p.L346R) were detected in our patient diagnosed as congenital heart disease with heart valve abnormalities. The novel frameshift mutation results in a complete loss of 48 amino acids in the Ig‐like domain and causes the formation of a putative protein product which might affect the IDUA enzyme activity.

**Conclusions:**

A novel compound heterozygous *IDUA* mutation (c.1815dupT, p.V606Cfs51^*^) was found in a Chinese MPS I family. The identification of the mutation facilitated accurate genetic counseling and precise medical intervention for MPS I in China.

## Graphical abstract

A novel compound heterozygous *IDUA* mutation in a Chinese family. Sequential analysis and homology modeling are efficient approaches for gene mapping and mutation identification.

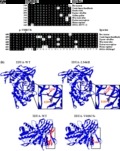

## 1 INTRODUCTION

Mucopolysaccharidosis type I (MPS I, OMIM#252800), an autosomal alpha‐L‐iduronidase (IDUA, EC 3.2.1.76) deficiency, resulting from *IDUA* (NG_008103.1) mutations affects the enzyme activity of alpha‐L‐iduronidase. So far, more than 100 *IDUA* causative mutations have been reported (Human Gene Mutation Database, http://www.hgmd.org/), which lead to progressive dysfunction of several organs and three MPS I phenotypic subtypes: Hurler syndrome (severe form), Hurler/Scheie syndrome (intermediate form), and Scheie syndrome (mild form) (Beck et al., 2014; Beesley et al., 2001; Bie et al., 2013; Champion et al., 2010).

The alpha‐L‐iduronidase enzyme, required for the degradation of glycosaminoglycans (GAGs) dermatan and heparan sulfate, was coded by *IDUA* (Chen et al., 2017). The ubiquitously expressed *IDUA* (located at position 4p16.3, NCBI reference sequence NM_000203.5) contains 14 exons and 13 introns, has 19 kb in length encoding the 653 amino acid IDUA polypeptide (Clarke et al., 2017).

Mutations in *IDUA* have an estimated incidence of one in 100,000 live births (Hopwood & Morris, 1990). To date, 100 mutations in the *IDUA* have been identified (Human Gene Mutation Database) (http://www.hgmd.org; 2019.9). Genetic mapping of MPS I patients will contribute to the identification of specific genotypes, genotype–phenotype associations, and also for new therapeutic options foundation. The aim of this study was to report a novel *IDUA* variation causing MPS I in a Chinese consanguineous family.

## 2 METHODS

### 2.1 Ethical compliance and patients' information

This study was approved by the local Ethics Committees and written informed consent was obtained from all patients participating in the study. This MPS I Chinese family was geographically localized in Linfen, Shanxi Province. The patients have been subjected to clinical and physical examinations and all the medical records were reviewed and evaluated.

### 2.2 Genotyping

**Figure 1:**
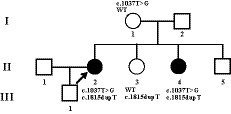
Pedigree of patients from the Chinese MPS I family. The patient involved in this study is pointed by an arrow. Filled circles indicate affected females. The carrier statuses of certain family members are shown

Whole‐exome sequencing (WES), variant calling, and filtering were performed as described previously (Kim et al., 2015). Basically, 3–5 ml blood samples were obtained from the patient and her family members (Figure 1). Genomic DNA was extracted from peripheral blood samples as described previously (Kwak et al., 2016). High‐throughput sequencing (HTS) and whole‐exome capture were carried out by the Veritas Genetics (Hangzhou, China). Shortly, the Illumina HiSeq2500 platform and SeqCap EZ MedExome Target Enrichment Kit (Agilent, California, USA) were used to capture the whole exomes as 150 bp paired‐end runs. The sequencing reads were aligned to the UCSC hg19 (human reference genome). Short insertions and deletions (InDels) and single‐nucleotide variants (SNVs) were filtered according to the functional annotation using rare disease NGS analysis platform (inhouse cloud‐based) with builds in public databases (1,000 Genomes, dbSNP, ESP, OMIM, Clinvar) as previously described (Kim et al., 2015).

The *IDUA* mutation was detected by Sanger sequencing using the primer pairs for exon 8 (Fw: 5ʹ‐CAACACCACCTCCGCCTTCCCCT‐3ʹ, Rv:5ʹ CAGCCCCATGGCCGTGAGCAC‐3ʹ) and exon 13 (Fw: 5ʹ‐CAGGTGCCTGTGGACATACGAG‐3ʹ, Rv: 5ʹ ‐TGAGGCCCAAGAATGGGGT‐3ʹ).

### 2.3 Bioinformatics analysis

Several in silico tools have been employed for the pathogenicity prediction: Mutation Taster, PolyPhen‐2, PROVEAN, and SIFT. Amino acid residue alteration in evolutionary conservation was compared across different species. The crystal structures of IdoA‐bound human IDUA (SMTL ID: 3w81.1) are available (Lacombe & Germain, 2014). Structural models of the mutant IDUA were built by the homology modeling programs Swiss‐Model (http://swissmodel.expasy.org) and the effects of mutated region were mimicked. PDB‐Viewer software was used to display the structures as described (Kwak et al., 2016).

## 3 RESULTS

### 3.1 Clinical features and family history

In this study, all patients were recruited from a Chinese family from Shanxi Province (Figure 1). At the age of 2, the patient (II‐2) developed abnormal joints development of hands, feet, and limbs. The flexion of metacarpophalangeal joints could not be stretched. At the age of 11, she developed blurred vision and gradually decreased vision. So far, only light perception has been found in both eyes.

The patient II‐4 developed similar symptoms but even earlier onset than patient II‐2. However, the son (9‐year‐old, III‐1) of the patient II‐2 does not show similar symptoms so far.

The patient was diagnosed as congenital heart disease, abnormal mitral valve development, mitral stenosis (severe), anterior mitral valve prolapses and insufficiency (moderate), patent ductus arteriosus (tubular), pulmonary hypertension (moderate), pericardial effusion (moderate); hydrocephalus, bilateral temporal arachnoid cyst; congenital spinal stenosis; congenital glaucoma, corneal gray degeneration, corneal staphyloma.

### 3.2 Sequencing results

To determine the disease‐causative gene mutation in this family, a WES on genomic DNA was performed. Totally, 120,421 genetic mutations, containing 14,807 nonsynonymous changes, occurred at the coding sequence or the canonical dinucleotide of the splice site junctions. The mutations were filtered to exclude variants with MAF （minor allele frequency） >0.01 observed in publicly available databases, such as Clinvar, HGMD, 1,000 Genomes, and gnomAD. Subsequently, compound heterozygous mutations were identified in *IDUA* associated with the disease phenotype.

**Figure 2:**
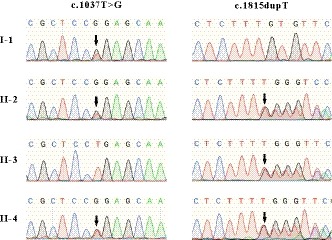
Sanger sequencing analysis of the genomic DNA from indicated patients. The gene mutation is shown by black arrow. The c.T1037G (p.L346R) mutation of *IDUA* in exon 8 was detected in I‐1, II‐2, and II‐4. The novel heterozygous single base insertion (c.1815dupT, p.V606Cfs51^*^) in exon 13 of *IDUA* was detected in II‐2, II‐3, and II‐4

Appropriate segregation in the extended pedigree was confirmed by Sanger sequencing (Figure 2). Sanger sequencing was performed to identify the molecular characterization of the *IDU*A (NG_008103.1, NM_000203.5, NP_000194.2) for individuals I‐1, II‐2, II‐3, and II‐4 (Figure 1). A novel heterozygous single base insertion in exon 13 of *IDUA* (g.17057dupT, c.1815dupT, p.V606Cfs51^*^) was identified in II‐2, II‐3, and II‐4, which is predicted to cause a frameshift in the coding sequence. And, the compound heterozygous mutation in exon 8 of *IDUA* (c.T1037G, p.L346R), which was reported previously (Li et al., 2019; Maita et al., 2013), was detected in II‐2 and II‐4 with MPS phenotype. For the healthy patients, we only got the blood from the patient's mother (I‐1) and the heterozygous mutation c.T1037G was detected.

The novel *IDUA* mutation (c.1815dupT, p.V606Cfs51^*^) described in our study was not found in the Clinvar, HGMD, 1,000 Genomes, gnomAD, and available literatures. Both mutations (c.T1037G, p.L346R and c.1815dupT, p.V606Cfs51^*^) were predicted to be disease‐causing by Mutation Taster, PolyPhen‐2, PROVEAN, and SIFT.

### 3.3 Molecular analysis

**Figure 3:**
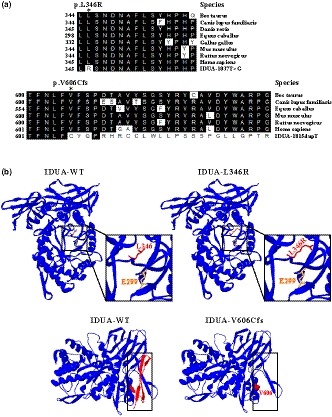
Analysis of *IDUA* mutation. (a) Evolutionary conservation of amino acid residues altered by c.T1037G (p.L346R) and c.1815dupT (p.V606Cfs51^*^) across different species. NCBI accession numbers are as follows: Bos Taurus (NP_001179665); Canis lupus familiaris (XP_538109); Danio rerio (XP_001923689); Equus caballus (XP_001492699); Gallus gallus (XP_420183); Mus musculus (NP_038491); Rattus norvegicus (NP_001102290); Homo sapiens (NP_000160). (b) The mutant proteins were structured by Swiss‐Model online software compared to the wild‐type. Ribbon representation of the human IDUA and map of the studied variant localization obtained by homology modeling analysis. The wild‐type and mutant monomers are shown in blue. Amino acid L346 and enzyme activity site E299 are shown as red and yellow stick, respectively. And, amino acid V606 is shown as red ball. The lost Ig‐like domain resulted from the frameshift mutation (c.1815dupT, p.V606Cfs51^*^) is shown by red ribbon in wild‐type

Evolutionary conservation analysis of amino acid residues showed that the impaired amino acid residues L346 and V606‐P642 were highly evolutionary conserved among IDUA proteins from different species (Figure 3a), indicating these mutations were likely disease‐causing predisposing to MPS I. There are three domains in human IDUA: a (β/α)_8_ TIM barrel domain (residues 42–396), a β‐sandwich domain (residues 27–42 and 397–545) with a short helix‐loop‐helix (residues 482–508), and an Ig (immunoglobulin)‐like domain (residues 546–642) (McKusick, Howell, Hussels, Neufeld, & Stevenson, 1972; Poletto, Pasqualim, Giugliani, Matte, & Baldo, 2018) (Figure 3b).

An especially high frequency of *IDUA* mutations in p.L346R was found in China. Among 57 Chinese patients with MPS I, the percentage of p.L346R mutant in *IDUA* alleles was accounted for 12.3% (14/114), indicating that most Chinese patients with MPS I were carrying p.L346R. The amino acid change from leucine to arginine at position 346 located in TIM barrel domain increased the length of the side chain which might affect the enzyme activity due to the approximate position of the active site E299 (McKusick et al., 1972; Poletto et al., 2018) (Figure 3b). Furthermore, functional analysis showed that p.L346R did not cause an apparent reduction in *IDUA* mRNA or protein level but result in highly reduced IDUA activity (0.4% of normal activity) in transfected COS‐7 cells (Saito, Ohno, Maita, & Sakuraba, 2014).

The novel frameshift mutation c.1815dupT (p.V606Cfs51^*^) resulted in a complete loss of 48 amino acids in the Ig‐like domain causing the formation of a putative amino acid product (Figure 3a,b). Ig‐like domains are one of the most common protein modules found in animals, occurring in a variety of proteins. Domains with an Ig‐like fold can be found in many diverse proteins in addition to immunoglobulin molecules.

## 4 DISCUSSION

MPS I, a rare autosomal genetic disorder, is caused by alpha‐L‐iduronidase deficiency due to *IDUA* alterations with globally variable phenotypic distribution. One in every 100,000 live births were affected (McKusick et al., 1972; Scott et al., 1995) and so far, more than 200 *IDUA* variations have been found in HGMD with majority of them being missense mutations. In this study, we recruited a patient who was misdiagnosed as congenital heart disease and showed no improvement after routine treatment. To accurately diagnose this disease with complex phenotypic heterogeneity, WES was performed and a novel heterozygous single base insertion in exon 13 of *IDUA* (c.1815dupT, p.V606Cfs51^*^) and a known compound heterozygous mutations in exon 8 of *IDUA* (c.T1037G, p.L346R) were identified in this MPS I Chinese family.

Evolutionary conservation analysis of amino acid residues showed that amino acid residues L346 and V606‐P642 are most highly evolutionary conserved among IDUA proteins from different species, indicating these mutations were likely pathological (Figure 3a). Human IDUA contains a (β/α)_8_ TIM barrel domain (residues 42–396), a β‐sandwich domain (residues 27–42 and 397–545) with a short helix‐loop‐helix (residues 482–508), and an Ig (immunoglobulin)‐like domain (residues 546–642) (McKusick et al., 1972; Poletto et al., 2018) (Figure 3b).

Sequencing analysis revealed that this novel in‐frame insertion (c.1815dupT, p.V606Cfs51*) resulted in a complete loss of 48 amino acids in the Ig‐like domain causing the formation of a putative 51 amino acid product which terminated at the nineth nucleotide of the 3’ UTR (Figure 3b). The known variation (c.T1037G, L346R) has been confirmed to be disease‐causing with highly reduced IDUA activity in transfected COS‐7 cells (Saito et al., 2014).

A putative protein product, nonsense‐mediated mRNA decay, and even premature chain termination formation due to altered reading frame shift can be caused by the insertion/deletion type of variations (Teng, Wang, Hwu, Lin, & Lee‐Chen, 2000; Valstar et al., 2010). In this study, the known mutation of *IDUA* (c.T1037G, p.L346R) is pathogenic as previously reported, but the heterozygous single base insertion of *IDUA* (c.1815dupT, p.V606Cfs51^*^) is unknown. The absence of the Ig domain and the formation of the putative amino acid product might affect the enzyme activity of IDUA.

## 5 CONCLUSION

In MPS I, the accumulation of partially degraded GAGs in most organs and tissues resulted in a progressive multisystemic disease with a wide range of clinical manifestations. These include dysostosis multiplex, coarse facial features, corneal clouding, inguinal and umbilical hernias, dilated cardiomyopathy, visceromegaly, hearing loss, restrictive lung disease, valvular heart disease, airway obstruction, and cognitive and developmental delays (Wang et al., 2012). For disease with such complex phenotypic heterogeneity, WES will be a very useful tool to facilitate the diagnosis.

Taking together, our study expands the *IDUA* mutation spectrum and contributes to the recognition and accurate diagnosis of its impact on phenotypic expression in MPS I patients, which was previously misdiagnosed as congenital heart disease for almost a decade. With the benefit from hematopoietic stem cell transplantation and recent enzyme replacement therapy, early diagnosis and multidisciplinary management of MPS can significantly improve these patients' life quality.

## 6 ETHICAL APPROVAL AND CONSENT TO PARTICIPATE

This study was approved by The Second Hospital, Shanxi Medical University Ethics Committees, and written informed consent was obtained from all patients participating in the study.

## AVAILABILITY OF SUPPORTING DATA

Please contact author for data requests.

## CONSENT FOR PUBLICATION

All patients participating in the study have given the written informed consent.

## CONFLICT OF INTEREST

The authors declare no competing financial interests.

## AUTHOR CONTRIBUTIONS

YAZ performed the clinical investigations, PL participated in the data analysis and drafted the manuscript, YPZ and QHX carried out the molecular genetic studies, CL and ZHZ helped with the study coordination and proof read the manuscript. YXW proof read the manuscript and HX conceived, designed, and helped to draft the manuscript. All authors read and approved the final manuscript.
